# On the shedding of impaled droplets: The role of transient intervening layers

**DOI:** 10.1038/srep18875

**Published:** 2016-01-08

**Authors:** Christos Stamatopoulos, Thomas M. Schutzius, Christian J. Köppl, Nicolas El Hayek, Tanmoy Maitra, Jaroslav Hemrle, Dimos Poulikakos

**Affiliations:** 1 Laboratory of Thermodynamics in Emerging Technologies, Mechanical and Process Engineering Department, ETH Zürich, 8092 Zurich, Switzerland; 2 ABB Switzerland, Corporate Reasearch, 5405 Baden-Daetwill, Switzerland

## Abstract

Maintaining the non-wetting property of textured hydrophobic surfaces is directly related to the preservation of an intervening fluid layer (gaseous or immiscible liquid) between the droplet and substrate; once displaced, it cannot be recovered spontaneously as the fully penetrated Wenzel wetting state is energetically favorable. Here, we identify pathways for the “lifting” of droplets from the surface texture, enabling a complete Wenzel-to-Cassie-Baxter wetting state transition. This is accomplished by the hemiwicking of a transient (limited lifetime due to evaporation) low surface tension (LST) liquid, which is capable of self-assembling as an intervening underlayer, lifting the droplet from its impaled state and facilitating a *skating*-like behavior. In the skating phase, a critical substrate tilting angle is identified, up to which underlayer and droplet remain coupled exhibiting a pseudo-Cassie-Baxter state. For greater titling angles, the droplet, driven by inertia, detaches itself from the liquid intervening layer and transitions to a traditional Cassie-Baxter wetting state, thereby accelerating and leaving the underlayer behind. A model is also presented that elucidates the mechanism of mobility recovery. Ultimately, this work provides a better understanding of multiphase mass transfer of immiscible LST liquid-water mixtures with respect to establishing facile methods towards retaining intervening layers.

Water droplet mobility is important to the functionality of many applications such as self-cleaning processes (*Lotus effect*)^1^,^2^, condensation^3^,^4^ and freezing^5^,^6^,^7^,^47^. It is closely associated with inhibiting contact between the water molecules and the solid, which is achieved by the presence of an intervening fluid (gas or liquid) layer. An illustrative example of such a layer is the *Leidenfrost effect*, wherein a water droplet levitates on its own vapor above a hot surface (temperature much higher than the boiling point), and the droplet is rendered highly mobile^8^,^9^. A similar mechanism has been demonstrated with a droplet impacting on a sublimating carbon dioxide surface. The sublimated gas layer minimizes the adhesive forces between the droplet and the solid substrate resulting in full drop rebound and preventing freezing^10^. Likewise, for rough hydrophobic surfaces, due to the air pockets that are formed inside the asperities, the interaction between the liquid and substrate is constricted and a water droplet can easily roll off or rebound^11^.

An alternative approach for promoting droplet mobility can be sought by the formation of an intervening liquid layer (previous examples were gaseous layers). This can be achieved by utilizing low-surface tension liquids, with negligible solubility in water, to form an ultra-smooth, low-hysteresis lubricant layer infused into a micro/nano structured hydrophobic surface. This is the concept underpinning slippery liquid-infused porous surfaces (SLIPS) or liquid infused surfaces (LIS)^12^,^13^. These surfaces are highly water repellent and maintain good performance under condensation and freezing conditions; despite the crucial role of an intervening layer in promoting droplet mobility, if it cannot be sustained through evaporation or other reasons, loss of the water repellent property will occur.

For self-cleaning applications on textured, hydrophobic surfaces, a loss in droplet mobility—as a result of a Cassie-Baxter-to-Wenzel transition—will lead to a corresponding loss in the self-cleaning property^14^. Regarding an impacting droplet, the absence or constriction of the gaseous layer will result in drop penetration into the asperities of the surface roughness and its final impalement. Furthermore, in condensation processes, a loss in mobility may cause the onset of filmwise condensation, which is detrimental to heat-transfer performance^15^,^16^,^17^.

Additionally, concerning LIS and SLIPS, although initial results are promising in terms of their droplet mobility, it has been shown that potential drainage of the suffusing lubricant may occur due to gravitational effects^18^. Regarding their anti-frost and anti-icing performance^18^,^19^, it has been pointed out that the process of condensation freezing and frost formation on such surfaces can lead to lubricant migration out of the structure with consequent loss of the intervening lubricant layer over time^19^ and its icephobic behavior.

Due to the impact of the intervening layer loss in the aforementioned processes, recovery mechanisms should be explored. Specifically, for the case of a rough hydrophobic surface where a water droplet transitioned from a Cassie-Baxter wetting state to a Wenzel one, previous research has demonstrated the ability to reverse this wetting transition through a variety of directly active processes, *i.e.*, electrowetting^20^, electrolysis^21^,^22^, pressure^23^,^24^,^25^, vibrations^26^,^27^,^28^, and magnets^29^. However, generic, semi-passive approaches involving solely the interplay of the interfacial energies, in the absence of external fields (which are limited to liquids responsive to them) or input of mechanical work, deserve further consideration, since these approaches are facile and energy efficient in their implementation.

This study focuses on the fundamental aspects of solid-LST liquid-water interactions and will provide insight into the mechanism that regulates the water droplet remobilization on tilted substrates. We demonstrate that an impaled droplet (Wenzel state) can be rendered mobile again on a functional superhydrophobic surface by wetting the surface with a LST liquid, *i.e.*, hydrofluoroether (HFE), without damaging the surface. After remobilization, we investigate the dynamics of the droplet motion that slides on the formed HFE intervening layer which, with the contribution of inertial forces, can be replaced by a gaseous (air) intervening layer, leading finally the moving droplet to a traditional Cassie-Baxter state.

## Experimental Process

**Figure 1:**
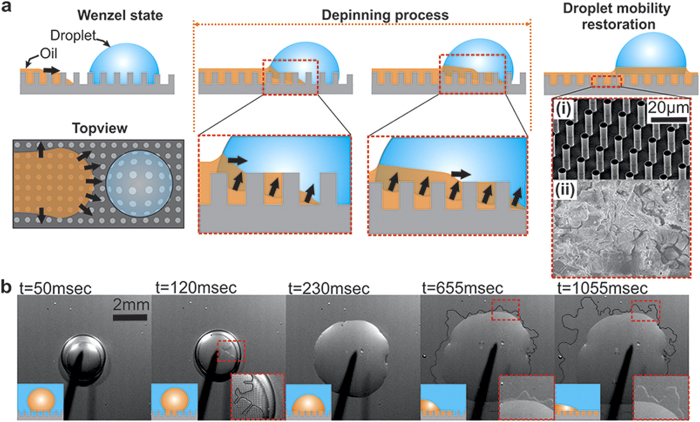
The role of a low surface tension liquid on the droplet wetting state. (**a**) Schematic showing how a water droplet can be depinned from a textured substrate by a hemiwicking LST liquid front (Wenzel-to-pseudo-Cassie-Baxter transition). Substrate is (i) a micropillar structured silicon-based SH substrate and (ii) an aluminum-based SH with random micro/nano roughness features. (**b**) Demonstration of a lubricant hemiwicking in a water environment, *i.e.*, displacing liquid water from the surface texture, which is one of the requirements for the process described in (**a**) to be favorable.

For the experimental procedure, two different hydrophobic substrates were used as depicted in Fig. 1(a): A micropillar structured silicon-based surface (Fig. 1(a(i))) and a randomly structured aluminum-based surface with hierarchical roughness (Fig. 1(b(ii))). In the first section of Results and Discussion, the wetting behavior of HFE on the aforementioned smooth and textured surfaces in air and water environments is presented. The HFE hemiwicking ability, a crucial feature for the shedding of impaled water droplets, was evaluated and compared to other apolar liquids which exhibit low surface tension, namely, FC-770 and hexane (see Supplementary Discussion Section 1). It should be noted that all tested liquids were immiscible in water.

In the following sections, a study of the droplet sliding behavior, after depinning by hemiwicking HFE, was performed on a home-built tilting stage viewed from the top and side with a high-speed camera illuminated with a cold light source; tilting angles (*α*) are measured with respect to the horizontal. Analysis of droplet position was carried out by post-processing with ImageJ enabling the calculation of velocity and acceleration. Water droplets were impaled into the surface texture by first covering the surface texture with HFE, then placing the droplet (8–12 μL) with a pipette. Under room temperature conditions, the HFE vaporized relatively fast, and as it withdrew from the surface texture, the interface between HFE and water was drawn into the surface asperities, resulting in an impaled state. Once impaled, the surface was tilted to the desired angle and then a known volume of HFE (~0.6 μL) was deposited in its vicinity, so that the hemiwicking and de-impalement processes could be initiated.

## Results and Discussion

### The hemiwicking effect

As discussed before, even on the best performing hydrophobic surfaces, after long-term exposure to droplet traffic, droplets may penetrate into the texture and eventually become pinned (Wenzel wetting state^30^). Figure 1a presents a hypothetical scenario where droplets have their mobility restored, through an intermediate lubricating process. For this to be successful, the lubricating liquid should exhibit hemiwicking behavior^31^ on the textured surface in both air and water environments. Modifying the analysis by de Gennes *et al.*^32^, this can be summarized by two different criteria: 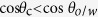 and 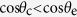, where *θ*_c_ is the critical contact angle defined by the following formula, 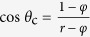, *r* is the ratio of the actual surface area to the projected one, *φ* is the liquid-solid wetting fraction, and 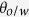 and *θ*_e_ are the intrinsic equilibrium contact angles of the LST liquid on the untextured substrate in a water and air environments, respectively (see Supplementary Discussion Section 1)^32^. Of the tested liquids FC-770 hexane and HFE, the latter exhibited hemiwicking behavior (see Supplementary Fig. S1) both in water and air environments fulfilling the two criteria mentioned previously. This was confirmed for the case of the silicon surface, where it was found that 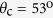, and the HFE intrinsic contact angles were 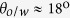 and 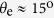. Figure 1b shows the behavior of the HFE on the silicon-based hydrophobic surface, where the condition 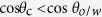 is satisfied (see Supplementary Discussion Section 1).

The de-impalement time-scale is governed by the time it takes for the liquid meniscus to flow underneath the droplet. If the HFE liquid is placed at some distance away from the water droplet and, after it penetrates the texture it reaches the water droplet as a film, then we can treat this flow as a fringe film. The above mentioned time-scale in this case is 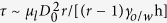
33, which for a millimetric water droplet (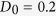 cm), a typical hydrophobic surface (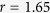, 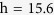 μm), and a fluorinated solvent (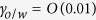 N m^−1^; 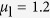 cP) yields 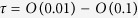
s. Therefore, we should expect de-impalement to be practically an instantaneous process (see Supplementary Discussion Section 1).

### The Wenzel transition after depinning

**Figure 2:**
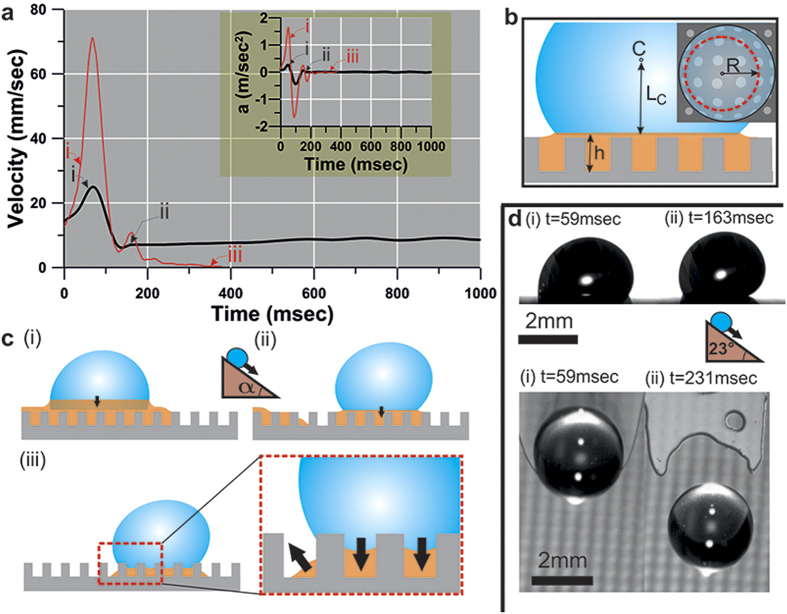
The pseudo-Cassie-Baxter wetting state of the droplet and its final transition to a Wenzel state. (**a**) Droplet velocity and acceleration, a, vs. time, measured from the droplet cross sectional centroid “C” as shown in (**b**). After detachment from the liquid layer on the silicon-based surface (black line) a terminal velocity U = 8.6 mm/sec is reached and acceleration is practically zero. For the aluminum-based surface (red line), the droplet transitions from phase (i) to (iii) and results in a loss of droplet mobility. (**b**) Various geometrical considerations for the droplet skating on the thin LST liquid film (**c**) Illustration of the sliding behavior of water droplet after mobilization with the use of a low surface tension liquid (HFE). (**d**) Side and top views of water droplets before (i) and after (ii) detachment from the bulk HFE liquid at inclination of 23°.

After the droplet is de-impaled from the surface texture through use of HFE and resides in a pseudo-Cassie-Baxter wetting state, it is further mobilized by tilting the surface. With a liquid (HFE) intervening layer formed on the two surfaces under the droplet, even a small tilting angle of α < 5° is sufficient to induce droplet motion due to the low contact angle hysteresis, 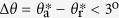 34,35, where 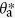 and 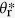 are the apparent advancing and receding contact angles, respectively. Figure 2a shows the velocity and acceleration of the droplet versus time. These were calculated based on the recorded frames of the side-view and subsequently the distance travelled by the cross-sectional centroid of the droplet C (Fig. 2b).

For 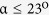, the water droplet sliding behavior on silicon-based surfaces exhibits two phases of motion. In *phase (i)*, as depicted in Fig. 2a (black line), it begins to slide down on the LST liquid coated substrate with 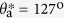, 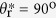, and 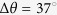 or 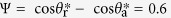 (Fig. 2c(i–ii), side view). After a certain period of acceleration, and after a maximum velocity is reached, its speed reduces significantly and rapidly. At this instant, the water droplet detaches from the bulk HFE layer (Fig. 2d, top view; see also Supplementary Video S1) retaining a small amount of HFE underneath it (Fig. 2c(ii)) and exhibits 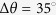 and 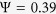 (Fig. 2d(ii), side view); this constitutes *phase (ii)* which allows the droplet to slowly “skate” on the surface. It is evident that the transition between *phases (i)* and *(ii)* is reflected in the change of the contact angle hysteresis as well (see Supplementary Video S2). During *phase (ii),* due to the entrapment of HFE the droplet skates on top of an HFE disk sustaining its pseudo-Cassie-Baxter wetting state and a terminal velocity is reached (see also Supplementary Video S2) which is attributed to a balance of forces (gravitational, viscous, surface tension), *i.e.*, 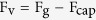, where F_g_ is the gravitational force exerted on a droplet with volume, F_cap_ is the capillary force exerted on the substrate, and F_v_ is the viscous force assuming that the sliding droplet is rolling and slipping^36^. Accounting for dissipation from motion inside the droplet, within the intervening fluid layer and the water-LST liquid-air contact line, and assuming that the LST liquid film resembles a Couette flow, one can estimate the terminal skating velocity U of the droplet with the following equation (see Supplementary Discussion Section 2 for full derivation):


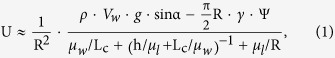


where *μ*_w_ and *μ*_l_ are the dynamic viscosity of water and the LST liquid, respectively, *ρ* and *γ* are the density and surface tension of water, respectively, *V*_*w*_ and R are droplet volume and contact disk radius, respectively, g is the acceleration due to gravity, h is the height of the silicon micropillars and L_c_ is the distance of the centroid C from the bottom of the droplet. Comparison between model and experiment yields that the model predicts satisfactorily the increasing trend of the terminal velocity with droplet size (see Supplementary Figure S6) and same order of magnitude velocities. However, the model provides smaller values compared to the experimental results, which is related to the overestimation of the capillary force (see also see Supplementary Discussion Section 2).

For the case of the aluminum-based substrate (red line, Fig. 2a), for tilt angles equal or less than 22°, the sliding droplet never reaches a terminal velocity and it transitions directly from *phase (i)* to *phase (iii)*, where *phase (iii)* is a complete loss of droplet mobility. This is a result of the stochastic surface roughness of the aluminum sample, which contains features with sharp edges of different heights that intensify the pinning effect (Fig. 1a(ii)). In combination with HFE’s volatility, and owing to the intermolecular forces exerted on water, while HFE is evaporating the droplet meniscus is drawn into the asperities of the rough substrate (Fig. 2c(iii)). The latter results in the pinning of the droplet and its final immobilization. The aforementioned sliding behavior of the droplet can change if additional inertia is given to the droplet as a result of increased α, as shown in the following section, and can cause the droplet to transition from a pseudo-Cassie-Baxter to a traditional Cassie-Baxter wetting state.

### The Cassie state restoration

**Figure 3:**
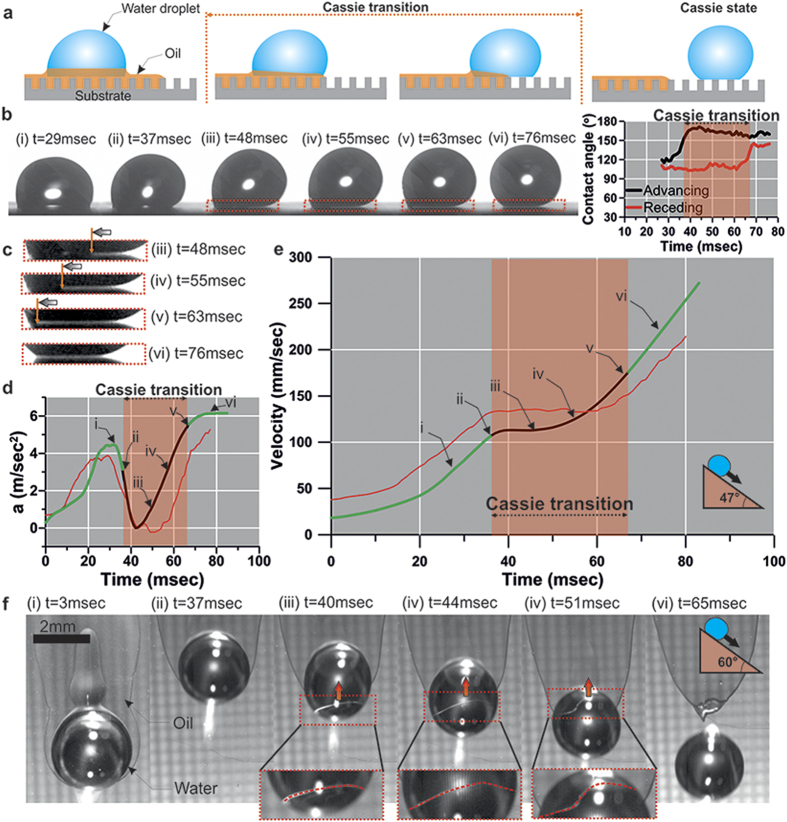
Transitioning from pseudo-Cassie to traditional Cassie-Baxter state. (**a**) Illustration of the sliding behavior of water droplet (10 μl) followed by its detachment from the liquid layer and final Cassie state restoration. (**b**) Side views of water droplets before (i) during (iii)–(v) and after (vi) detachment from the LST liquid film at inclination of 47°; Dynamic change of advancing and receding contact angle vs time (**c**) Magnifications showing the development of the air layer underneath the droplet. (**d**) Time-dependent acceleration of water droplet and (**e**) velocity vs. time for a droplet on a silicon-based (green-black line) and aluminum-based (red line) surface. (**f**) Top views of droplet motion depicting the motion of the LST liquid-water-air front for inclination 60°. Cases (i)–(vi) correspond to the states described in (**b–e**).

In the previous section, it was shown that for α < 23° (silicon-based surface), two outcomes are possible: the impaled droplet can be remobilized, finally reaching a terminal velocity, or it can re-impale the substrate. If α ≥ 29°, then the droplet sliding behavior deviates from the type of sliding observed previously (Fig. 3a). In particular, the droplet accelerates (Fig. 3b(i)), and when it reaches a critical velocity of 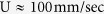 (acceleration 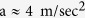; Fig. 3b(ii) and d), the front part of the droplet begins to detach from the HFE film. The part that detaches transitions to the traditional Cassie-Baxter wetting state (Fig. 3b(iii–v)) which expands upstream and finally the Cassie-Baxter wetting state is fully restored (Fig. 3b(vi)). The dynamic changes of the advancing and receding contact angles during this transition are shown in Fig. 3b (contact angle vs time). At the transition point, the advancing contact angle value increases from 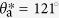 (Fig. 3b(i)) to approximately 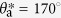 (Fig. 3b(iii–v)) and gradually drops to approximately 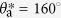. On the other hand the receding angle does not change significantly during the transition process remaining around 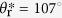 which indicates the presence of the intervening LST liquid film (Fig. 3b(i,ii) and d). After the completion of the transition, the receding contact angle increases to 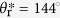 and the advancing contact angle remains 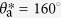, thus indicating the traditional Cassie-Baxter wetting state restoration (Fig. 3b(vi), see also Supplementary Video S3).

Furthermore, apart from the change of the advancing and receding contact angle, a white stripe between the water droplet and the SH substrate is observed (Fig. 3c), which is associated with the air entrapped into the asperities of the surface roughness. The presence of the air cushion in combination with the proper configuration of the light source enables light to pass underneath the droplet and renders possible its visualization^37^,^38^. Consequently, the development of the Cassie-Baxter state restoration can be clearly associated with the white stripe that propagates with time towards the upstream direction (Fig. 3c, see also Supplementary Video S3).

The process of detachment is accompanied with a temporary acceleration decrease (Fig. 3d,e, black line) to a minimum value 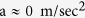 in the early stage at 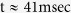. Subsequently, the droplet accelerates again reaching a constant value of 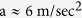 which corresponds to the acceleration of a water droplet sliding on a dry surface (see Supplementary Discussion, Section 3) indicating that it has been totally separated from the LST liquid layer. It should be noted that after detachment a slight increase of the acceleration is observed until stabilization. This can be attributed to the deformation of the droplet shape while it is separating from the HFE film, which simultaneously affects the circular shape of contact line and respective capillary force exerted on it. Exploiting the transparent property of water together with employing proper illumination conditions, the HFE-water-air line underneath the droplet was visualized (Fig. 3f) revealing additional information regarding the separation process. The aforementioned line viewed from the top at various time instants demonstrates a wavy profile, which changes its geometrical features along the propagation process. It is noticeable that between time instants t = 51 msec and t = 65 msec, a slight change in the droplet shape occurs as already mentioned before (see Supplementary Video S4).

For the case of aluminum-based substrate and for α ≥ 34° (Fig. 3d,e, red line), the sliding of the droplet demonstrates slightly different behavior. The droplet reached the critical velocity of 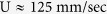
(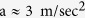) before the onset of detachment, denoting that more inertia is required for the Cassie-Baxter transition to occur.

### Kinetic energy of detachment

**Figure 4:**
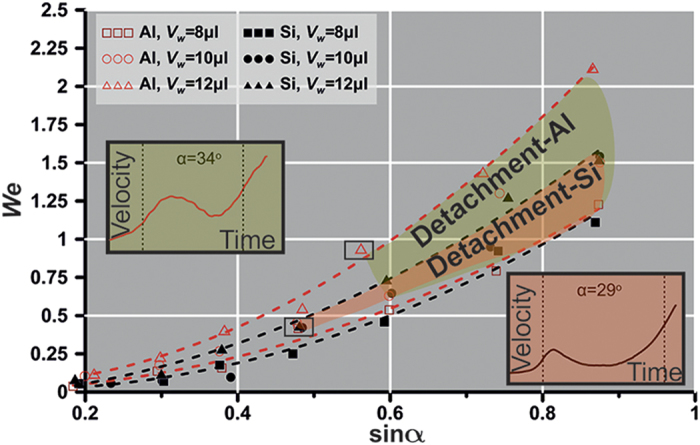
*We* vs sinα for aluminum and silicon surfaces. *We* corresponds to the maximum velocity observed before detachment or droplet impalement. For aluminum and silicon surfaces the onset of detachment is observed at 34° and 29° respectively. Three different droplet sizes were investigated 8 μl, 10 μl and 12 μl. Dashed lines (red for aluminum-based surface, black for silicon-based surface) are curve fittings of the experimental data corresponding to 8 μl (lower dashed lines) and 12 μl (upper dashed lines).

The deviation in the sliding behavior between the aluminum-based and silicon-based surfaces is also illustrated in Fig. 4 for three droplet sizes i.e. 8 μl, 10 μl and 12 μl. To facilitate the discussion, we define the Weber number as 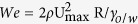 where U_max_ is the maximum velocity observed before the droplet reaches a terminal velocity or re-impales the substrate (see section ‘Wenzel transition after depinning’) or detaches from the liquid intervening layer (see section ‘The Cassie state restoration’) and R is the radius of the droplet contact line. It is indicated that for the case of aluminum, *We* is higher in comparison to the silicon surface. Consideration of the three droplet sizes allows the determination of detachement zones; it is shown that detachment zone of the aluminum-based substrate is wider compared to the silicon-based surface. This denotes that excess of kinetic energy in the range 48–118% (Fig. 4) is required for the detachment to take place at the aluminum surface due to its random roughness that enhances the pinning effect. Note that onset of detachment phase was observed at 34° (*We* = 0.94) and 29° (*We* = 0.42) tilt angles for the aluminum and silicon surfaces, respectively for 12 μl droplet. With decreasing size the detachment occurs in higher tilting angles, namely for droplet size 8 μl transition to traditional Cassie occurs at 37° for both surfaces. The onset at these tilt angles is typically represented by the corresponding velocity vs time graphs (Fig. 4, inset), where the velocity shows a considerable drop during the transition phase (region between the dashed lines). This is the critical state where the droplet marginally overcomes the pinning of the surface (Fig. 2c) and transitions to Cassie-Baxter (Fig. 4).

### Modelling Cassie state transition

**Figure 5:**
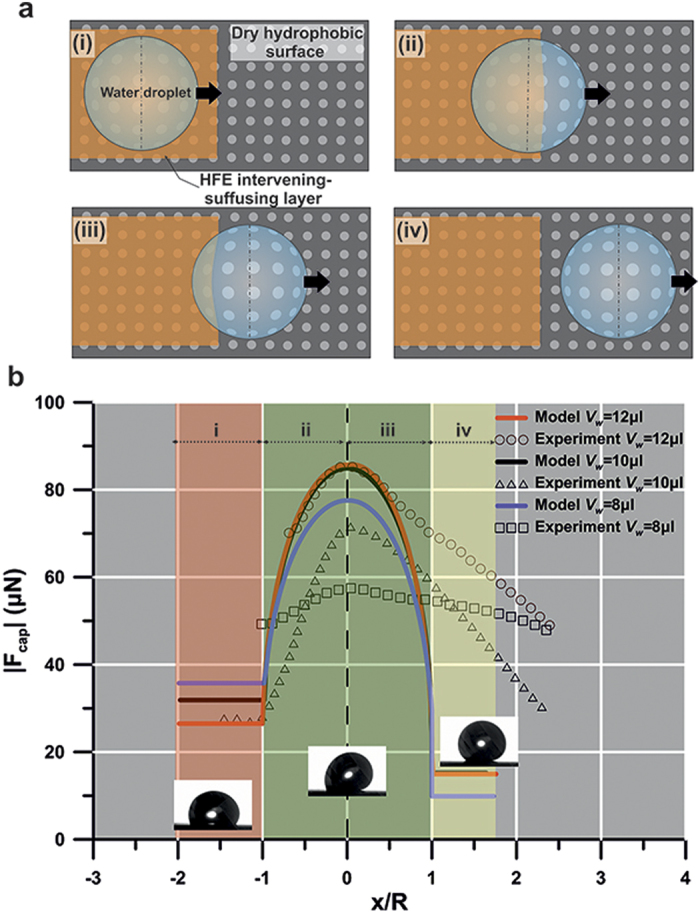
Model of the droplet’s sliding transition where the liquid (HFE) intervening layer switches to a gaseous (air) one. (**a**) Illustration of the different phases a sliding water droplet of three different sizes (8 μl, 10 μl, 12 μl) undergoes. During stage (i) the droplet is sliding on the HFE decorated surface. Subsequently it enters the dry hydrophobic surface ((ii)–(iii)), and finally it is separated from the HFE intervening layer being in contact totally with the dry hydrophobic substrate. (**b**) Capillary force |F_cap_| vs. normalized position x/R of RP along the transition phase (experiment and model). Tilt angle was α = 47°.

In order to elucidate features of the separation mechanism, a model is constructed that estimates the capillary force, F_cap_, exerted by the substrate. To simplify, it is assumed that the droplet preserves the circular shape of its contact line through the entire process—as shown in Fig. 5a—and that the water-HFE-air front is straight (not corrugated). Moreover, this front is considered to be stationary, contrary to the real case, where a slight motion of the front is observed (Fig. 3f). Thus, two regions are defined with different wettability characteristics, namely, a LST liquid coated and a dry hydrophobic surface. The model is based on the calculation of the infinitesimal dF_cap_ (due to interfacial interactions) acting on the differential length of the 3-phase contact line dS (see Supplementary Discussion Section 4 for a complete derivation)^32^,^39^,^40^:


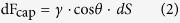


where *θ* is the contact angle of the droplet corresponding to the length *dS* normal to the force. For the calculation of F_cap_, the droplet motion was divided in 4 stages (Fig. 5a). In stage (i) the droplet slides on the HFE intervening layer, in stage (ii) it gradually leaves the HFE layer until the first hemicircle of the contact disk enters the dry hydrophobic surface, in stage (iii) the second half of the contact disk enters the dry hydrophobic region and finally in stage (iv) the entire droplet slides on the dry hydrophobic region.

By comparing the model with the experimental data at α = 47°, it is noted that the capillary force behavior is similar (Fig. 5b) for three different droplet sizes (8 μl, 10 μl and 12 μl). When the droplet centroid is approaching the wettability step, the magnitude |F_cap_| of the capillary force is maximized (acceleration minimized, Fig. 3d) opposing its motion. In the case of a wettability gradient, this capillary force is in the direction of the less hydrophobic region^41^,^42^, which in our case is the LST liquid coated surface. Initially, the water droplet is on the hemiwicked hydrophobic surface exhibiting 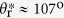 and 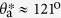 (10 μl droplet). During the time interval when the contact disk enters and advances in the dry hydrophobic region (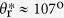 and 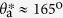, 10 μl droplet), |F_cap_| increases reaching a maximum value (Fig. 5b) at the instant where the contact disk is evenly divided between the two different wettability regions. Subsequently, this force magnitude decreases as the droplet is leaving the liquid intervening layer behind. It is found that the trend predicted by the simplified model is in satisfactory qualitative agreement with the experiments. It should be noted that discrepancies between experiments and model were also observed. In particular, apart from the 12 μl droplet, where both model and experiment predicted a maximum resisting forces of 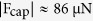, for the case of 10 μl and 8 μl the experimentally obtained maximum resisting forces were by 20% and 33% smaller, respectively, compared to the model; for α = 47° these values are 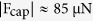 (model), and 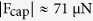 (experiment) for a 10 μl droplet, 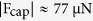 (model), and 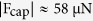 (experiment) for a 8 μl droplet. The differences are attributed to inherent errors of contact angle measurements and the fact that in reality the contact angle varies between the receding and advancing contact angle values along the droplet contact circumference^43^,^44^. Furthermore, for the case of the experimentally determined force, the respective bell-shape curve is broader than the diameter of the contact line, contrary to the case of the model. This can be attributed to the mobility of the wettability step due to the hemiwicking effect during the process of detachment, which results in a longer transition region. In addition, when the center of the contact disk is leaving the wettability step (Fig. 5b stage (iii)) the model is not describing adequately the process of force decrease, which can be associated with the deformation of the droplet shape deviating the contact line from a circle^45^.

### Conclusions

It was demonstrated that an impaled water droplet on a hydrophobic surface can be de-impaled and undergo a transition to a pseudo-Cassie-Baxter wetting state with the spontaneous formation of an intervening low-surface-tension (LST) liquid layer. Therefore, liquid intervening layers constitute a semi-passive and facile method of mobilizing impaled droplets. Two cases were studied: a well controllable silicon-based surface for fundamental studies of the sliding behavior following the de-impalement process and an aluminum-based surface directly relevant to industrial applications. It was demonstrated that by fabricating a superhydrophobic surface from a mechanically robust material used in practical aplications (aluminum) and by employing a scalable technique to impart excellent water repellent properties, satisfactory shedding behavior of impaled droplets could be achieved. It was shown that for substrate tilt angles equal or greater than 29° (silicon surface) and 34° (aluminum surface), the droplet is able to transition from pseudo-Cassie state to traditional Cassie-Baxter state and the LST liquid intervening layer is replaced by an air layer. In order to acquire additional information about the transition phase, a model was developed, which confirmed that the presence of the capillary force is a result of the different wettability regions experienced by the drop. Despite the fact that the aluminum surface exhibits a stronger pinning effect originating from its stochastic roughness, by increasing the critical tilt angle the droplet mobility can be sustained. These findings are relevant to the performance of liquid suffused surfaces, where the droplet mobility can be sustained/restored even when local regions of liquid depletion exist

## Methods

### Surface fabrication: Aluminum

To create a hydrophobic coating on an aluminum substrate^46^, a mirror-like polished aluminum substrate (AW 1085) with a size of 20 mm × 20 mm was immersed in NaOH aqueous solution (1% w/w) at 45 °C for 15 minutes to remove the aluminum oxide layer that covering its surface. Then, the sample was etched with an FeCl_3_ aqueous solution (1 mol L^−1^) at 25 °C for 7.5 minutes. After this step the surface becomes superhydrophilic and has a microstructured morphology.

The second step comprises of functionalizing the substrate in order to reduce its free energy by forming a self-assembled monolayer (SAM). Prior to SAM coating, the sample was immersed in a H_2_O_2_ aqueous solution (30% w/w) for 30 minutes to re-oxidize the surface for enhancing SAM formation. Then, the substrate was immersed in a perfluorodecyltrichlorosilane (FDTS) – hexane solution (0.05% v/v). To promote FDTS hydrolization, a very thin water layer was formed over the surface before immersion. Functionalization took place at 7 °C for 1 hour and 45 minutes followed by rinsing in hexane and heating at 120 °C for 45 minutes. After the second step, the sample was hydrophobic and displayed advancing contact angles >150° and contact angle hysteresis <10°. To increase performance, the hydrophobic Al samples were further treated with a third step. This consisted of dip coating in a PDMS (50 mg, 10% curing agent)–FDTS (100 μL)–THF (Tetrahydrofuran, 10 mL) solution and curing it at 135 °C for 30 minutes. At the end of the last step the sample exhibited durable hydrophobic characteristics. The measured advancing and receding contact angles for water droplets were 168.4° and 164.8°, respectively^46^.

### Surface fabrication: Silicon

To fabricate a hydrophobic micropillar surface, a p-type (100) silicon wafer was photolithographically patterned (Karl Suss MA6; AZ1505 positive photoresist) and the exposed wafer regions were etched in a SF_6_ and C_4_F_4_ plasma (Bosch process in Alcatel AMS 200 machine). Subsequently, the photoresist film was stripped, the wafer was diced (2 cm × 2 cm chips), and after standard cleaning the chips were coated with FDTS (1H, 1H, 2H, 2H-Perfluorodecyltrichlorosilane; 96% Alfa Aesar) in an *n*-hexane solution. The micropillar surface had the following properties: pillar diameter, d = 4.3 μm; pillar pitch, 18 μm; pillar height, 15.6 μm (liquid/solid wetting fraction, *ϕ* = 4.5%). The measured advancing and receding contact angles for water droplets on the hydrophobic surface were 164° and 149°, respectively.

### Surface characterization

Wettability characterization of the hydrophobic surfaces was carried out by measuring advancing and receding contact angle values with the sessile drop method. Microliter-scale volumes of water were dispensed and withdrawn through a flat tipped needle placed near the substrate; the droplet volumes were controlled with a syringe pump (New Era, NE1000-E). Contact angle measurements were made with images captured with a home-built backlit image acquisition setup, which consisted of a camera (Thorlabs DCC1545M, CMOS) fitted with a zoom lens (Thorlabs Zoom 7000 TV Lens MVL7000). Morphological characterization of the surfaces was done with a Zeiss ULTRA 55 scanning electron microscope (SEM). For the aluminum-based samples, to facilitate visualization, they were first sputter coated with a layer of Pt.

To investigate the hemiwicking behavior of hydrofluoroether (HFE; 3M™ Novec™ 7100) on textured silicon and aluminum in a water environment, first the samples had to be evacuated of all vapor/gas. To achieve this, the samples were immersed in a deionized water bath (EMD Millipore, Direct-Q 3), placed into a desiccator (Kartell) and subjected to low-vacuum conditions until boiling was observed. Once the sample was returned to ambient pressure, the liquid impaled the surface. If air/vapor bubbles still remained, then a water jet was used to dislodge them from the surface. Subsequently, HFE droplets were dispensed onto the aluminum or silicon textured surfaces through a needle placed near the substrate (~10 μL). A high-speed camera (Phantom v9.1) was then placed overhead to view the hemiwicking process from a “top-view” perspective.

## Additional Information

**How to cite this article**: Stamatopoulos, C. *et al.* On the shedding of impaled droplets: The role of transient intervening layers. *Sci. Rep.* **6**, 18875; doi: 10.1038/srep18875 (2016).

## Supplementary Material

Supplementary Information

Supplementary Video S1

Supplementary Video S2

Supplementary Video S3

Supplementary Video S4
