# Supplementary material for: On the shedding of impaled droplets: The role of transient intervening layers
Source: Sci Rep. 2016 Jan 8;6:18875. doi: 10.1038/srep18875 (PMC4705525; doi:10.1038/srep18875)
Supplement: Supplementary Information [file srep18875-s1.doc]

Supplementary Discussion

On the shedding of impaled droplets: The role of transient intervening layers

Christos Stamatopoulos^a^ , Thomas M. Schutzius^a^ , Christian J. Köppl^a^ , Nicolas El Hayek^a^ , Tanmoy Maitra^a^ , Jaroslav Hemrle^b^ and Dimos Poulikakos^a,1^

^a^Laboratory of Thermodynamics in Emerging Technologies, Department of Mechanical and Process Engineering, ETH Zurich, 8092 Zurich, Switzerland; and ^b^ABB Switzerland, Corporate Reasearch, 5405 Baden-Daetwill, Switzerland

^1^Correspondence and request for materials: [dpoulikakos@ethz.ch](mailto:dpoulikakos@ethz.ch)

Contents

[1. Wenzel-to-pseudo-Cassie-Baxter wetting transitions 3](#__RefHeading___Toc424480802)

[2. Derivation of droplet terminal velocity 5](#__RefHeading___Toc424480803)

[3. Droplet acceleration: The role of substrate type 8](#__RefHeading___Toc424480804)

[4. Derivation of the droplet capillary force 8](#__RefHeading___Toc424480805)

[Supplementary Figures 12](#__RefHeading___Toc424480806)

[Supplementary Videos 19](#__RefHeading___Toc424480807)

# Wenzel-to-pseudo-Cassie-Baxter wetting transitions

For this wetting transition to be spontaneous (Wenzel-to-pseudo-Cassie-Baxter), that is oil displacing water impaled in the surface texture, it should be thermodynamically favorable. (The following is a modified analysis from de Gennes *et al.*^1^). This can be written as

| , | **S****1** |
| --- | --- |

where $r$ is the ratio of true surface area to projected surface area, $\varphi$ is the water-solid wetting fraction, and the subscripts ‘o’, ‘w’, and ‘s’ refer to oil, water, and solid, respectively. By utilizing Young’s relation to define the intrinsic contact angle for an apolar droplet on a solid surface in a water environment, $\cos\theta_{\text{o/w}}=\left(\gamma_{\text{ws}}-\gamma_{\text{os}}\right)/\gamma_{\text{ow}}$, we see that Equation S1 becomes

| $\frac{1-\varphi}{r-\varphi}<\cos\theta_{\text{o/w}}$. | **S****2** |
| --- | --- |

This inequality mirrors the relation obtained for a droplet to be in a superhydrophilic wetting state (hemiwicking) on textured surface. ^1^ In this case, the strategy for achieving an apolar liquid droplet in a super-wetting state under-water (apparent contact angle, $\theta^{*}\approx0$°) is clear: functionalize the solid surface to achieve a relatively low value of $\theta_{\text{o/w}}$, and then texture the surface properly to maximize $r$ and minimize $\varphi$. In the case of the surface utilized in this study, $r=1.65$ and $\varphi=0.04$; therefore, the critical angle ($\theta_{\text{c}}$) for the transition to a hemiwicking state is $\cos\theta_{\text{c}}\approx0.60\Rightarrow\theta_{\text{c}}=53$°. For apolar liquid droplets to display such oleophilic behavior under-water, the surface should be rendered hydrophobic. This is readily achieved by fluorosilane deposition on the silicon surface.

The results of such surface texturing and chemical modification are shown in Figure S1, where for liquids having sufficiently low values of $\theta_{\text{o/w}}$ (measured as an advancing angle, $\theta_{\text{a}}$), superoleophilic behavior is observed ($\theta^{*}\approx0$°). For the liquids that did not display hemiwicking behavior ($\theta^{*}>0$°), their apparent contact angle value most closely follows that described by the Wenzel equation as, ^2^ $\cos\theta^{*}=r\cos\theta_{\text{o/w}}$, indicating that the liquids had enough affinity for the surface in order to impale it, but still not sufficient to hemiwick. The surfaces that had the superoleophilic property under-water are shown in Figure S2.

Most of the above discussion was on equilibrium wetting states; however, it did not give any insight into the dynamics of the super-wetting process. To better understand this, it is instructive to compare the hemiwicking behavior of HFE (the liquid that displayed hemiwicking behavior under-water) in both open-air and under-water. Figure S3 presents an image sequence (time in sec) of an HFE droplet hemiwicking on a superhydrophobic surface (; $\left[d,p,\text{h}\right]=\left[4.3,18,15.6\right]$μm) in open air and Figure S4 for the same conditions but in a water environment and a larger droplet. There are two main differences in the hemiwicking cases: 1) The shape that the hemiwicking front forms as it displaces air or water; 2) the speed that the meniscus advances with. The distance that the meniscus has advanced from the bulk droplet on a textured surface can be estimated from a Washburn-style equation as ^3–5^

| $L\sim\left(\frac{r-1}{r}\frac{\gamma\text{h}}{\mu}\right)^{1/2}t^{1/2}\propto C_{0}t^{1/2}$ , |  |
| --- | --- |

where $L=R_{\text{f}}-R_{\text{b}}$ (see inset in Figure S5 for a description of this variable), $R_{\text{f}}$ is the radius of the fringe of the droplet (meniscus), $R_{\text{b}}$ is the radius of the bulk of the droplet rising above the penetrating film, $\gamma$ is surface tension, $\text{h}$ is the pillar height, and $\mu$ is the viscosity of the advancing fluid. For a well-ordered surface/geometry, these parameters represent a pre-factor constant ($C_{0}$), and the position of the meniscus in time should follow as $L\propto t^{1/2}$. ^3^

Figure S5 presents a plot of $L$ vs. $t$ for HFE flowing within a micropillar surface in air and water environments. It is clear that the hemiwicking speed of HFE is measurably faster in an air environment vs. water; however, they are still comparable. For the air environment, the pre-factor should theoretically be $C_{0}\approx0.9$cm s^-0.5^ ($\text{h}=15$μm, $\gamma=\gamma_{\text{lv}}=16.2$ mN m^-1^, $\mu=1.2$ cP). From Figure S5 we see that $C_{0}\approx0.40$cm s^-0.5^ which is of the same magnitude, indicating that the hemiwicking behavior of HFE is similar to that of water on a superhydrophilic surface. ^3^ Due to the asymmetric way that the meniscus advances within the surface texture in a water environment, quantifying $L$ is non-trivial. While true, we can track one point of the meniscus to estimate the velocity, and this is plotted in Figure S5. It is obvious that the early stage dynamics are dramatically different in the two cases —air and water environments facilitate fast and slow meniscus speeds, respectively. Based on this, if the goal is to utilize an HFE droplet to de-impale a millimetric scale water droplet through hemiwicking in an air and water environment, then the process should happen in ~1s.

# Derivation of droplet terminal velocity

During *phase (ii)* of droplet sliding, which corresponds to a droplet gliding along a lubricating layer of HFE, it is observed that after a certain amount of time a terminal velocity is reached due to a balance of forces, *i.e.*, $\text{F}_{\text{v}}\text{=F}_{\text{g}}\text{-F}_{\text{cap}}$, where $\text{F}_{\text{g}}$ is due to gravity, $\text{F}_{\text{cap}}$ is the capillary force which opposes droplet motion for the case of a dry surface, and $\text{F}_{\text{v}}$ is due to viscosity (assuming that the sliding droplet is rolling and slipping).^6^ Accounting for dissipation from motion inside the droplet, within the suffusing layer, and the water-LST liquid-air contact line, and assuming that the suffusing film resembles a Couette flow, one can estimate the terminal velocity of the droplet (Fig 2c, main text). The force due to gravity acting on the droplet is calculated by the following equation:

| $\text{F}_{\text{g}}\text{=}\rho V_{w}g\text{sinα}$, | S3 |
| --- | --- |

where $V_{w}$ is the volume of the water droplet, $\rho$ is the density of water , $g$ is the acceleration due to gravity, and $\text{α}$ is the substrate tilting angle (with respect to the horizontal).

The force $\text{F}_{\text{cap}}$ is the force due to surface tension and in this case resists motion of the droplet (contact angle hysteresis): ^6–10^

| $\text{F}_{\text{cap}}=\left(\pi/2\right)\text{R}\gamma(\cos\theta_{\text{r}}^{\text{*}}-\cos\theta_{\text{a}}^{\text{*}})$, | **S4** |
| --- | --- |

where $\text{R}$ is the radius of contact between the droplet and substrate, and $\theta_{\text{a}}^{\text{*}}$ and $\theta_{\text{r}}^{\text{*}}$ are the apparent advancing and receding angles of the droplet on the surface. The force $\text{F}_{\text{g}}-\text{F}_{\text{cap}}$ is then balanced by $\text{F}_{\text{v}}$, and is the sum of three terms:^11,12^ 1) The motion inside the droplet $\text{F}_{\text{vw}}$; 2) the motion within the LST liquid $\text{F}_{\text{vl}}$; 3) and the existence of a water-oil-air contact line $\text{F}_{\text{va}}$. Namely, $\text{F}_{\text{v}}$ is given by the following expression

$\text{F}_{\text{v}}=\text{F}_{\text{vw}}+\text{F}_{\text{vl}}+\text{F}_{\text{va}}$. **S5**

The first term can be defined as

| $\text{F}_{\text{vw}}\approx\mu_{\text{w}}\text{UR}^{2}/\text{L}_{\text{C}}$ , | **S6** |
| --- | --- |

where $\mu_{\text{w}}$ is the viscosity of water, $\text{U}$ is the velocity of the droplet centroid (point $\text{C}$), and $\text{L}_{\text{C}}$ is the vertical distance of point $\text{C}$ from the substrate (Fig 2d, main text). Employing a similar approach for the existence of the lubricating film we can write

| $F_{\text{vl}}\approx\mu_{\text{l}}\text{U}_{\text{l}}\text{R}^{2}/\text{h}$, | **S7** |
| --- | --- |

where $\mu_{\text{l}}$ is the oil viscosity, $\text{h}$ is the height of the silicon micropillars, and $\text{U}_{\text{l}}$ is the velocity in the lubricating film. Assuming that the motion inside the lubricating film resembles a Couette flow and by equalizing shear stresses due to the water and oil flow, we can write

| $\text{U}_{\text{l}}=\text{U}\left(1+\frac{\mu_{\text{l}}\text{L}_{\text{C}}}{\mu_{\text{w}}\text{h}}\right)$ , | **S8** |
| --- | --- |

and Equation S7 takes its final form:

| $\text{F}_{\text{vl}}\approx\frac{\text{UR}^{2}}{\text{h}/\mu_{\text{l}}+\text{L}_{\text{C}}/\mu_{\text{w}}}$ . | **S9** |
| --- | --- |

Finally $\text{F}_{\text{va}}$ is approximated by the following equation:

| $\text{F}_{\text{va}}\approx\mu_{\text{l}}\text{UR}$. | **S10** |
| --- | --- |

During its motion the droplet reaches a state where,

$\text{F}_{\text{v}}=\text{F}_{g}\text{-F}_{\text{cap}}$, **S11**

and its velocity is constant. Substituting Equations S3-S6 and S9-S10 into S11 and rearranging yields:

| $\text{U}\approx\frac{1}{\text{R}^{2}}\frac{\rho V_{\text{w}}g\sin\alpha-\frac{\pi}{2}\text{R}\gamma\left(\cos\theta_{\text{r}}^{\text{*}}-\cos\theta_{\text{a}}^{\text{*}}\right)}{\mu_{\text{w}}/\text{L}_{\text{C}}+\left(\text{h}/\mu_{\text{l}}+\text{L}_{\text{C}}/\mu_{\text{w}}\right)^{-1}+\mu_{\text{l}}/\text{R}}$ , | **S12** |
| --- | --- |

where $V_{\text{w}}$ is the entire volume of the droplet. $V_{\text{w}}$ is estimated assuming that the shape of the droplet is a spherical cap as^13^

| $V_{\text{w}}=\frac{\pi}{3}\text{R}_{\max}^{3}\left(2+\cos\theta\right)\left(1-\cos\theta\right)^{2}$ , | **S13** |
| --- | --- |

where $\theta=\left(\theta_{\text{a}}^{\text{*}}+\theta_{\text{r}}^{\text{*}}\right)/2$ and $\text{R}_{\max}$ is the droplet radius of curvature, which can be related to the contact disk radius described before as $\text{R}=\text{R}_{\max}\sin\theta$ . In Figure S6 the predicted and experimental terminal velocity for three different droplet sizes namely 8μl, 10μl and 12μl and tilt angle 23^o^ on silicon-based substrate is shown. Both model and experiment provide the same orders of magnitude and similar trends showing that terminal velocity is increasing with droplet size. It is apparent that the proposed model underestimates the droplet terminal velocity, which we attributed to the approximate formula used, (**S4**), for the estimation of the pinning force^6–10^ that yields a larger droplet adhesion to the substrate.

# Droplet acceleration: The role of substrate type

Figure S7 presents a plot of droplet acceleration vs. $\alpha$ for the experimental cases of a droplet sliding on a superhydrophobic surface consisting of aluminum and silicon. Also plotted is the theoretical case (maximum acceleration) where the droplet slides on a surface without any friction ($\text{\textasciitilde}g\text{sinα}$). It is clear that in the case of droplet sliding on an aluminum substrate, the acceleration experienced by the droplet is significantly less than both the theoretical limit and the experimental case of a droplet sliding on silicon. This indicates that the pinning force is much greater for droplets sliding on the aluminum substrate, which we attribute to the inherently stochastic structure of the surface providing more opportunities for contact line pinning.

# Derivation of the droplet capillary force

To understand the maximum capillary force that must be overcome for the droplet to transition from a pseudo-Cassie-Baxter wetting state to a true one, we use a model based upon an infinitesimal capillary force $d\text{F}_{\text{cap}}$. It is due to interfacial interactions acting on the differential length of the contact line $dS$^1,15^:

| $d\text{F}_{\text{cap}}=\gamma\text{cos}\theta dS$, | **S14** |
| --- | --- |

where $\theta$ is the contact angle of the droplet corresponding to the length $dS$ normal to the force. For the case where the entire droplet stands only on the LST liquid suffused surface or the dry hydrophobic case (see Fig S8 (i) and (iv)), it is assumed that for the right hand side semicircle of the contact line, the contact angle is constant and equals to $\theta_{\text{a}}$whereas for the left hand side semicircle the contact angle equals to $\theta_{\text{r}}$. Physically, for droplets on textured or chemically heterogeneous surfaces, $\theta_{\text{a}}$ and $\theta_{\text{r}}$ would be apparent contact angles, i.e. $\theta_{\text{a}}^{\text{*}}$ and $\theta_{\text{r}}^{\text{*}}$. Analysing $d\text{F}_{\text{cap}}$ in two components, $d\text{F}_{x}$ parallel and $d\text{F}_{y}$ perpendicular to the direction of the motion, it is clear the sum of the latter is zero due to symmetry. Calculating $d\text{F}_{x}$yields:

| , | **S15** |
| --- | --- |

where index $i$ is ‘a’ that corresponds to contact line semicircle at which contact angle is $\theta_{\text{a}}$ or is ‘r’ that corresponds to semicircle at which contact angle is $\theta_{\text{r}}$. Consequently, the total capillary force for a droplet sitting entirely on oil or a dry hydrophobic surface is calculated as follows (see Fig S8 i and iv):

| $\text{F}_{\text{cap}}\text{=}\int_{0}^{\text{2π}} \gamma\text{R }\text{cos}\theta_{i}\text{ cosφ dφ=}-2\gamma\text{R}\left(\cos\theta_{\text{r,j}}-\cos\theta_{\text{a,j}}\right)$. | **S16** |
| --- | --- |

where $j$ is an index that can be either 'o' which corresponds to the part of contact disc that is on the lubricant-decorated surface or 's' which corresponds to the part that is on the dry hydrophobic surface. It should be noted that $\text{F}_{\text{cap}}$calculated from S16 is negative, indicating that it is resisting the droplet motion. Utilizing the model for the cases (ii) or (iii) renders the calculation of $\text{F}_{\text{cap}}$ more complex. Assuming that part of the droplet enters the hydrophobic surface, and that the water-LST liquid-air line is at a distance x from its center (with the coordinate system (x’,y’) attached to the centre of the moving droplet) , then the contact disc line is divided into two zones: **Zone 1** , where and the contact angle hysteresis is $\Psi_{\text{o,s}}=\cos\theta_{\text{r,o}}-\cos\theta_{\text{a,s}}$, and **zone 2,** where or and where the contact angle hysteresis is either $\Psi_{\text{o,o}}=\cos\theta_{\text{r,o}}-\cos\theta_{\text{a,o}}$ for $0<\text{x}\leq\text{R}$(see Fig S8ii) or $\Psi_{\text{s,s}}=\cos\theta_{\text{r,s}}-\cos\theta_{\text{a,s}}$ for $-\text{R}\leq\text{x}\leq0$(see Fig S8iii). Accounting for the above and the symmetry about the droplet horizontal axis (x’), the total capillary force is

| , | **S17** |
| --- | --- |

where $j\equiv\text{o}$ for $0<\text{x}\leq\text{R}$and $j\equiv\text{s}$ for $-\text{R}\leq\text{x}\leq0$ (see Fig 5, main text).

References

1. Gennes, P. G. de, Brochard-Wyart, F. & Quere, D. *Capillarity and Wetting Phenomena: Drops, Bubbles, Pearls, Waves* (Springer, 2004).

2. Wenzel, R. N. Resistance of solid surface to wetting by water, *Industrial and Engineering Chemistry* **28,** 988–994 (1936).

3. Kim, S. J. *et al.* Liquid spreading on superhydrophilic micropillar arrays, *J. Fluid Mech.* **680,** 477–487 (2011).

4. Washburn, E. W. The Dynamics of Capillary Flow, *The Physical Review* **17,** 173–283 (1921).

5. Squires, T. M. & Quake, S. R. Microfluidics: Fluid physics at the nanoliter scale, *Reviews of Modern Physics* **77,** 977–1026 (2005).

6. Carre, A. & Shanahan, M. E. R. Drop Motion on an Inclined Plane and Evaluation of Hydrophobia Treatments to Glass, *The Journal of Adhesion* **49,** 177–185 (1995).

7. Extrand, C. W. & Kumagai, Y. Liquid Drops on an Inclined Plane: The Relation between Contact Angles, Drop Shape, and Retentive Force, *Journal of Colloid and Interface Science* **170,** 515–521 (1995).

8. Neckernuss, T. Wiedemann, S. Plettl, A. & Ziemann, P. Moving Water Droplets Over Nanoscaled (Super) hydrophobic Wettability Contrasts: Experimental Test of a Simple Model Describing Driving Forces, *Adv. Mater. Interfaces* **1,** 1300033, 1-8 (2014).

9. Yoshida, N. *et al.* Sliding behavior of water droplets on flat polymer surface, *Journal of the American Chemical Society* **128,** 743–747 (2006).

10. Sakai, M. *et al.* Image analysis system for evaluating sliding behavior of a liquid droplet on a hydrophobic surface, *The Review of scientific instruments* **78,** 045103, 1-5 (2007).

11. Mahadevan, L. & Pomeau, Y. Rolling droplets, *Physics of Fluids* **11,** 2449–2453 (1999).

12. Smith, J. D. *et al.* Droplet mobility on lubricant-impregnated surfaces, *Soft Matter* **9,** 1772–1780 (2013).

13. Quéré, D. Azzopardi, M.-J. & Delattre, L. Drops at Rest on a Tilted Plane, *Langmuir* **14,** 2213–2216 (1998).

14. Antonini, C. Carmona, F. J. Pierce, E. Marengo, M. & Amirfazli, A. General methodology for evaluating the adhesion force of drops and bubbles on solid surfaces, *Langmuir* **25,** 6143–6154 (2009).

Supplementary Figures


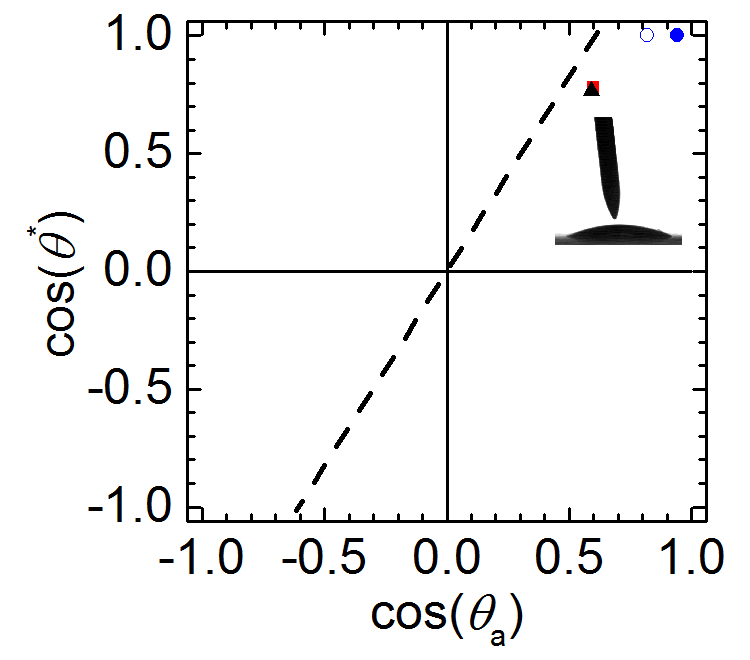


Figure S1. Wetting behavior of textured surfaces in a water environment. Plot of $\cos\theta^{*}$(textured surface) vs $\cos\theta_{\text{a}}$ (smooth surface) in an under-water environment for several apolar liquids, HFE (hydrofluoroether; ○ and ●), FC-770 (▲), and hexane (■), on silicon (filled symbols) and aluminum (unfilled symbols) substrates. Also included in is a line obtained from the Wenzel wetting model (- - -; $r=1.65$). Inset, image of an under-water $\theta^{*}$ measurement of HFE on a fluorosilane-treated silicon micropillar substrate Eventually, the angle becomes zero after some time.


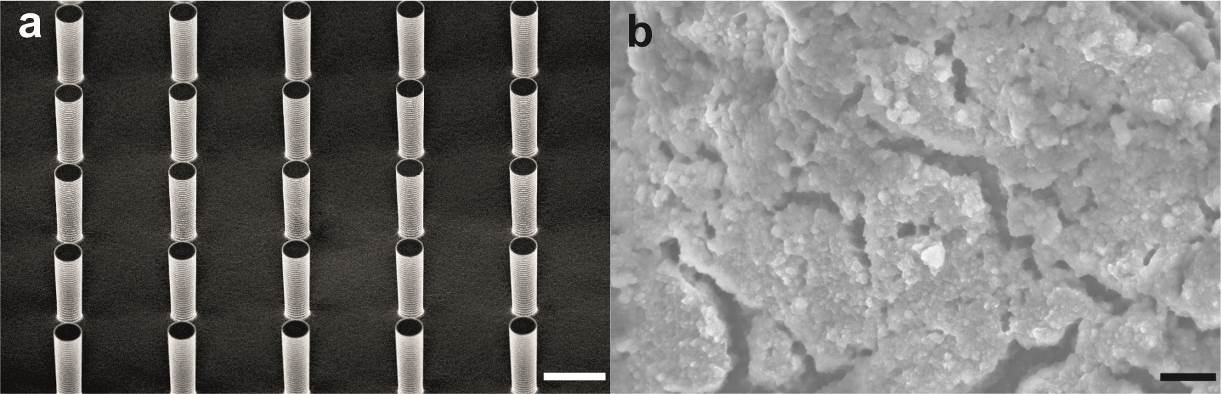


Figure S2. Micrographs of the surfaces used in this study. Scanning electron micrographs showing the superhydrophobic a, silicon micropillar and b, aluminum surfaces utilized in this study. The silicon micropillar surface has the following features: $d=4.3$μm (pillar diameter), $p=18$ μm (pitch), and $\text{h}=15.6$ μm (pillar height). Scale bars, a, 10 μm; b, 1 μm.


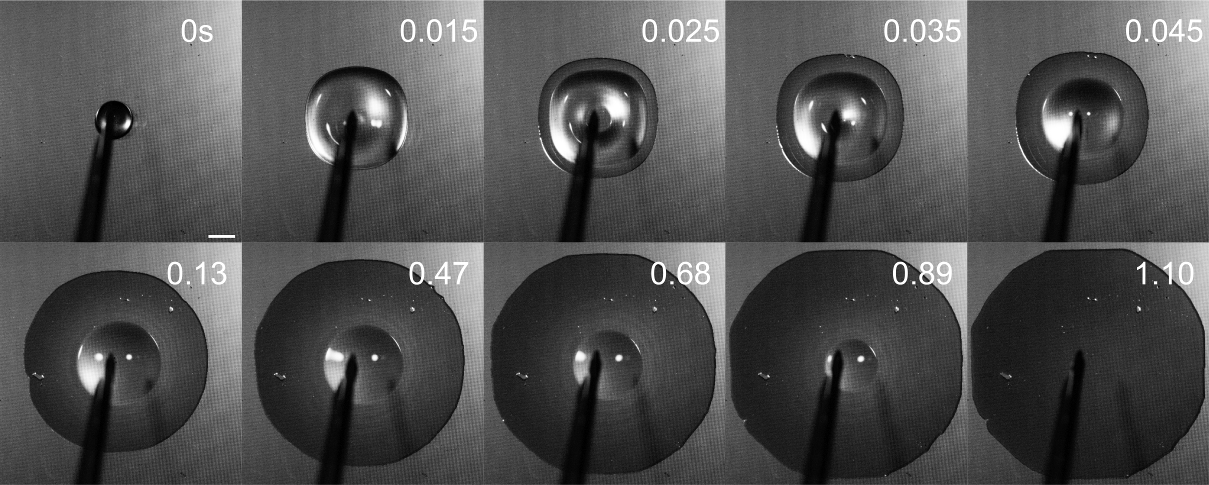


Figure S3. HFE hemiwicking behavior in an open-air environment. Image sequence showing an HFE droplet being placed with a needle onto a superhydrophobic silicon micropillar surface in open air demonstrating hemiwicking behavior. The scale bar is 1 mm. Inset is the time in seconds.


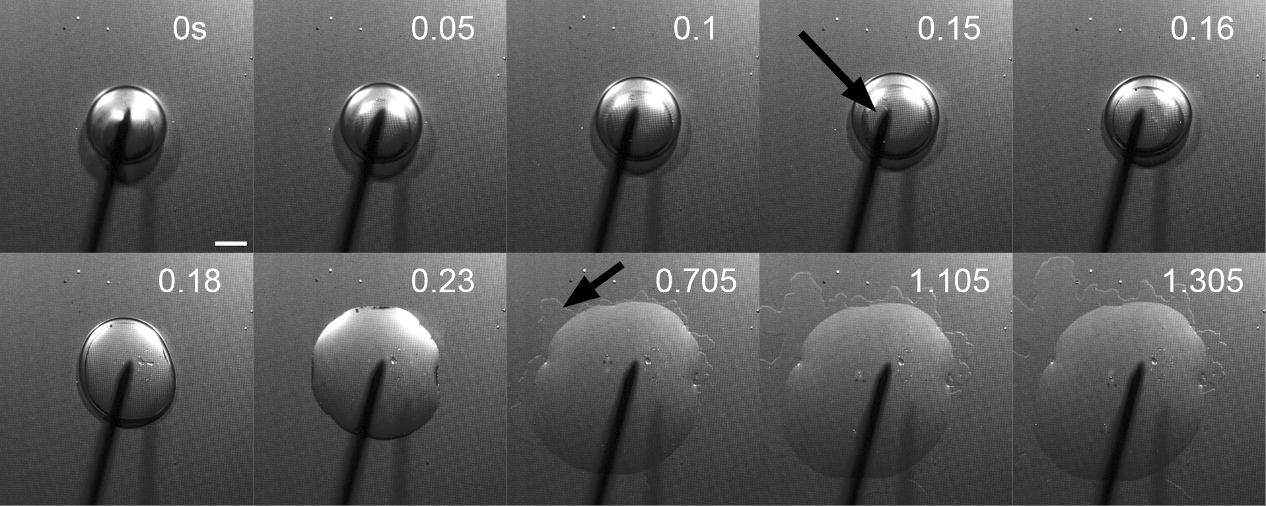


**Figure S4. HFE hemiwicking behavior underwater.** Image sequence showing an HFE droplet being placed onto a superhydrophobic micropillar surface in a water environment. The surface was filled with water prior to placing of the HFE droplet. The scale bar is 1 mm. The frames of note are at 0.15 s and 0.705 s. Arrows indicate HFE impaling the surface texture (0.15s) and initiating hemiwicking (0.705s).


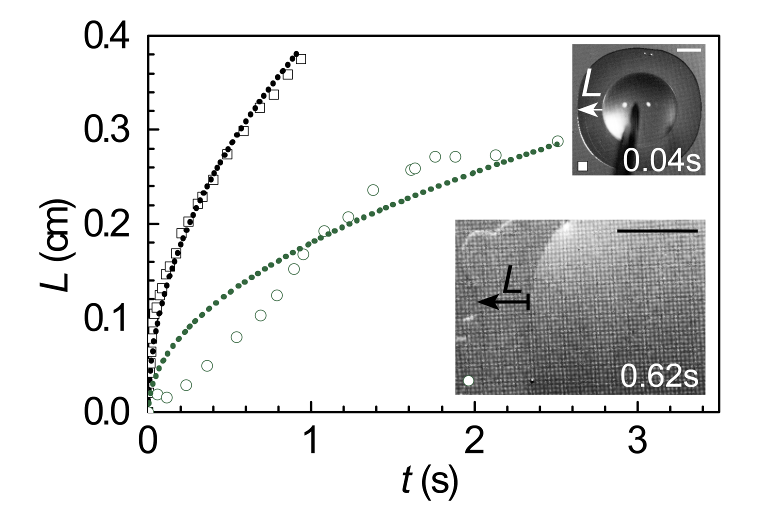


Figure S5. HFE meniscus behavior within microtexture for open-air and under-water environments. Plot of $L=R_{\text{f}}-R_{\text{b}}$ after hemiwicking has initiated on a superhydrophobic surface in air (black squares) and water (green circles) environments for HFE. Lines of best fit are also given as $L\propto0.40t^{1/2}$ (- - -) and $L\propto0.18t^{1/2}$(- - -). Inset are images of the two processes. Scale bars are 1.0 mm.


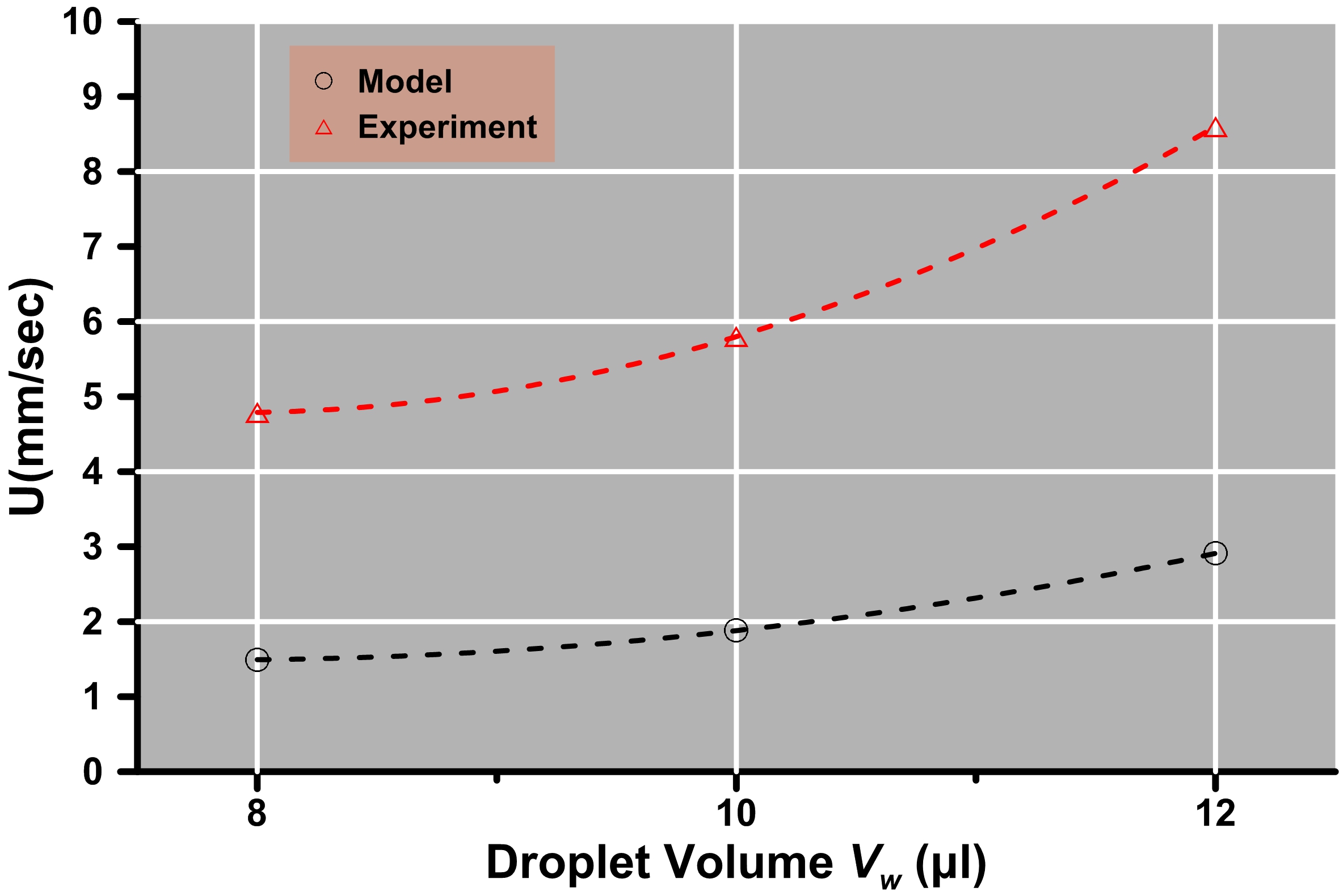


**Figure S6. Terminal velocity vs droplet size on silicon for tilt angle α=23°.** Predicted and experimental values are compared for three different droplet sizes , namely 8μl, 10μl and 12μl.

Figure S7. Acceleration of water droplet on dry aluminum-based (black circles) and silicon-based (red triangles) surface for a range of tilt angles α=13°-60°. Acceleration in both cases is lower than gravitational acceleration ($\text{F}_{\text{g}}\text{=ρV}_{\text{w}}\text{gsinα}$) which is attributed to the pinning force of the substrate. It is noticeable that the capillary force is greater for the case of aluminum-based surface than the case of silicon-based surface.


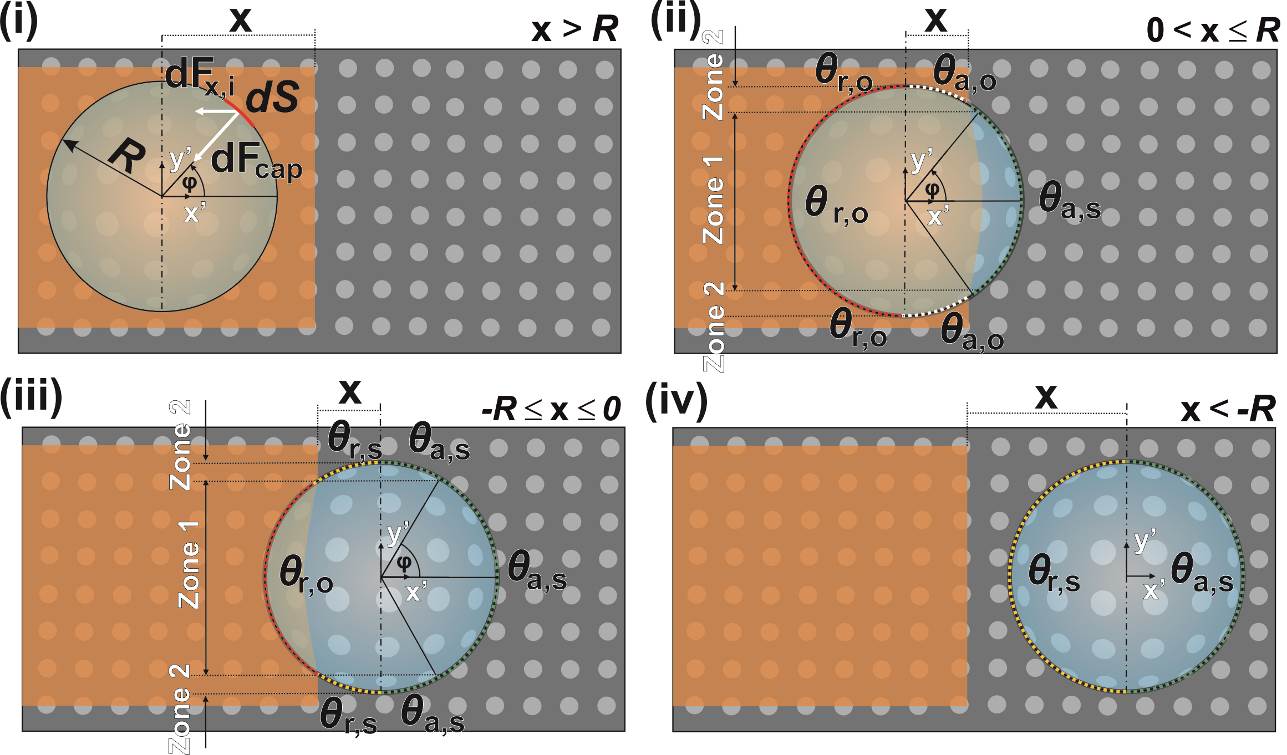


**Figure S8. Geometrical considerations and illustration of the different phases of the sliding motion of a water droplet.** During phase (i) the droplet is sliding on the HFE intervening layer. Subsequently it enters the dry hydrophobic surface ((ii)-(iii)), and finally detaches from the HFE intervening layer being in contact totally with the dry hydrophobic substrate.

#

# Supplementary Videos

**
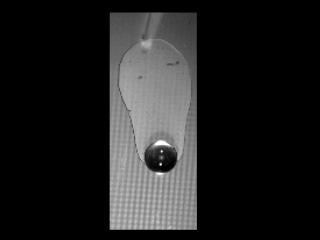
**

**Video S1**. Top view of inclined micropillar silicon-based SH surface ($\alpha=23$°). Water droplet detaches from the bulk LST liquid that covers the silicon substrate and finally reaches a terminal velocity. Recording frame rate 1000 fps; Playback speed $\times$0.25.


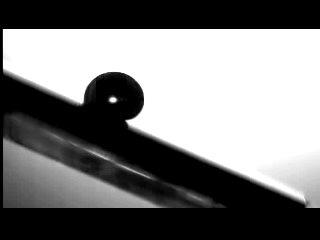


**Video S2.** Side view of inclined micropillar structured silicon-based SH surface ($\alpha=23$°). Water droplet detaches from the bulk LST liquid that covers the silicon substrate and finally reaches a terminal velocity. Recording frame rate 1000 fps; Playback speed $\times$0.25.


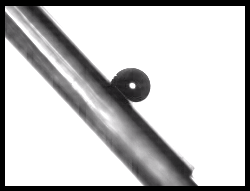


**Video S3**. Side view of inclined micropillar silicon-based SH surface ($\alpha=47$°). The water droplet gains adequate inertia so that it transitions to Cassie-Baxter state. Recording frame rate 1000 fps; Playback speed $\times$0.025.


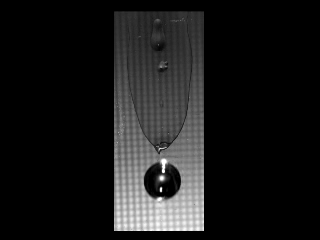


**Video S4**. Top view of inclined micropillar silicon-based SH surface ($\alpha=60$°). The water droplet gains adequate inertia so that it transitions to Cassie-Baxter state. Recording frame rate 1000fps; Playback speed $\times$0.025.
